# Minimal invasive management for untreated high-grade renal trauma and its complication: A case report

**DOI:** 10.1016/j.ijscr.2024.110175

**Published:** 2024-08-14

**Authors:** Anastasia Pearl Angeli, Soetojo Wirjopranoto, Yufi Aulia Azmi, Antonius Galih Pranesdha Putra, Kevin Muliawan Soetanto

**Affiliations:** a Faculty of Medicine, Universitas Airlangga, Surabaya, Indonesia; b Department of Urology, Faculty of Medicine Universitas Airlangga – Universitas Airlangga Academic Hospital, Surabaya, Indonesia; c Department of Health Sciences, University of Groningen, University Medical Center Groningen, Groningen, the Netherlands; d Department of Immunology, Faculty of Medicine Siriraj Hospital, Mahidol University, Bangkok, Thailand

**Keywords:** Renal trauma, Urinoma, Complications, Urinary diversion, Case reports, Infection

## Abstract

**Introduction and importance:**

Renal trauma is a common and associated complication of abdominal trauma. Although there is consensus that most high-grade injuries require surgical exploration, nonoperative management remains a viable approach. We aim to report case reports of four cases of nonoperative isolated high-grade blunt renal trauma in adults, followed by a literature review.

**Case presentation:**

A 22-year-old female presented to the emergency room (ER) with intermittent fever and associated symptoms of renal trauma, including persistent left flank pain, nausea, and vomiting. Three weeks earlier was diagnosed with left renal trauma AAST Grade IV. She was advised to go to the hospital but was refused admission. Then she came with intermittent fever, and a second abdominal computed tomography (CT) scan showed urinoma. The patient was managed with a Double J (DJ) stent and percutaneous drainage.

**Clinical discussion:**

Conservative management is the standard of care for hemodynamically stable patients with AAST grade I to III renal injury, regardless of the mechanism of efficiency. If perinephric fluid collection persists despite interventions such as ureteral stenting or percutaneous nephrostomy drainage, percutaneous drainage may facilitate healing and prevent or treat abscesses.

**Conclusion:**

Minimal invasive management DJ stent insertion and percutaneous drainage can be used as a treatment for untreated high-grade renal trauma and urinoma as its complication.

## Highlights

• Minimal invasive management due to high-grade renal trauma complication
• Conservative management of high-grade renal trauma needs total bed rest.
• High-grade renal trauma needs clinical and laboratory evaluation.

## 1 Introduction

Renal trauma accounts for approximately 50 % of all abdominal trauma cases and 5 % of total trauma cases [1]. Blunt renal trauma is the most prevalent type, comprising 71–95 % of all renal trauma cases [2]. Among blunt renal trauma cases, motor vehicle accidents (63 %) are the primary cause, followed by falls (43 %), sports-related incidents (11 %), and pedestrian accidents (4 %) [3]. In cases of blunt trauma, direct crushing of the kidney and hilar structures occurs, while avulsion injuries affecting the vascular structures of the hilum or the ureteropelvic junction (UPJ) are less common and usually result from sudden deceleration forces.

The American Association for the Surgery of Trauma (AAST) classification is the most widely used and validated categorization system for renal trauma [4]. This classification system has proven effective in predicting morbidity and the need for intervention, making it the preferred system for urological trauma management, along with hemodynamic consideration [5,6].

Significant renal injuries, ranging from grades II to V, are observed in only 5 % of renal trauma cases. Patients with stable hemodynamics AAST grade I to III renal injuries require a standard of care of non-operative approach, regardless of the underlying mechanism. While there is a consensus that most high-grade injuries require surgical exploration, non-operative management remains a possible approach, even for grade IV or V injuries, if carefully staged and selected [7,8].

There is a possibility that conservative therapy for high-grade renal trauma will fail. Kidneys can be saved in all stable patients with appropriate conservative management of Grade IV renal trauma, but it does require continuous clinical and radiological monitoring [9]. One possible complication is urinoma. Although the incidence of urinoma following renal trauma is thought to be minimal (<1 %), it rises with high-grade renal trauma [10]. Urine collection called urinomas is caused by a disruption in the urothelium, which can happen anywhere from the kidney to the bladder. They may be caused by a rupture or ischemia-induced urothelial necrosis, anastomotic leakage along the ureteroneocystostomy, or incisional damage [11].

This paper describes the management of failed conservative renal trauma accompanied by urinoma. This case report describes the management of high-grade renal trauma that was not treated properly accompanied by complications of fever, leukocytosis, and urinoma. The writing of this study follows the SCARE guidelines 2023 [12].

## 2 Case presentation

**Fig. 1:**
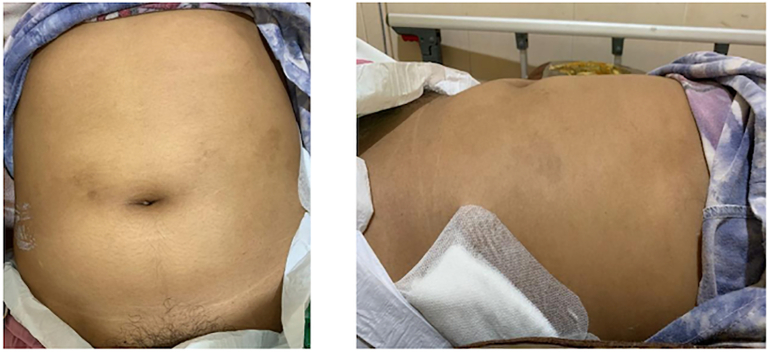
Clinical picture of the patient.

**Fig. 2:**
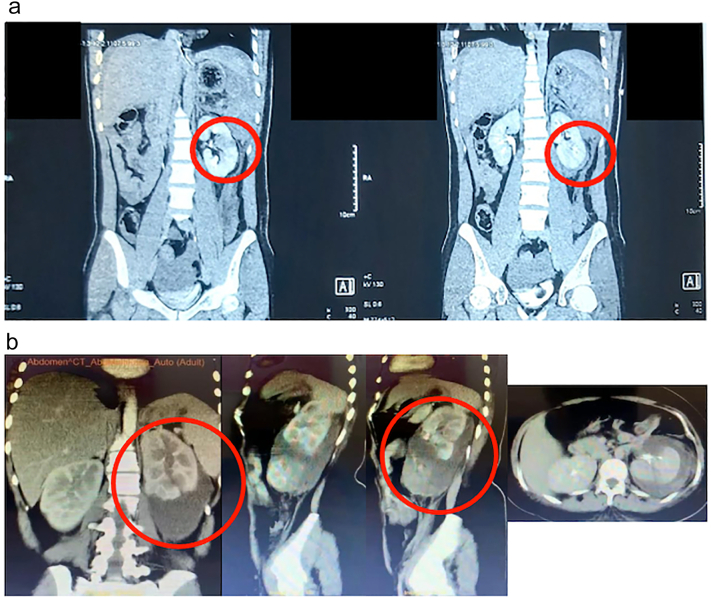
(a). First-time abdominal CT scan result. (b). Second-time abdominal CT scan result.

**Fig. 3:**
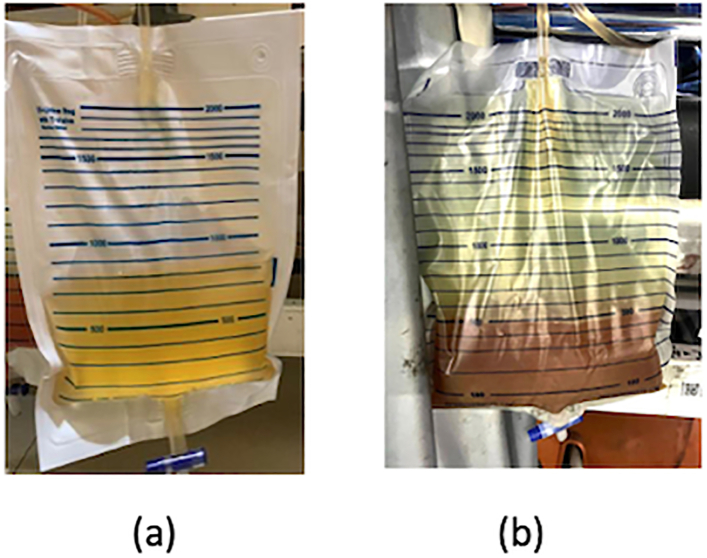
(a) Urine production via urethral catheter. (b) Percutaneous drainage production.

A 22-year-old female presented to the emergency room (ER) with intermittent fever and associated symptoms of renal trauma, including persistent left flank pain, nausea, and vomiting. Three weeks earlier, she was involved in a traffic accident, colliding with a motorcycle. She fell to the left side and the left flank hit the pedestrian. There was pain and bruising on the left flank after the accident; she was hemodynamically stable. She was already offered hospital admission, but she refused because she didn't have insurance. The doctor advised her to take a total bed rest of 14 days because the first abdominal CT scan result showed renal trauma AAST grade IV (Fig. 2(a)). When she came to the ER for the second time, a physical examination revealed a Temperature of 38.5 °C and left costovertebral angle (CVA) tenderness and ballottement of the left kidney (Fig. 1). Laboratory results showed leukocytosis 36.000 μL. Abdominal CT scan findings demonstrated parenchymal disruption, subscapular hematoma measuring 3 cm in diameter, and laceration of the pelvicocalyceal system (PCS), indicative of AAST grade IV renal trauma (Fig. 2b). The Retrograde Pyelography - Ureterorenoscopy (RPG-URS) revealed left meatus urethra externa (MUE) stenosis and lower pole contrast extravasation, indicating leakage. Hence, we implanted a 4.7 Fr DJ stent. We also performed percutaneous drainage of the urinoma on the left flank using a pigtail, resulting in pus-mixed urine amounting to 450 cm^3^/24 h (Fig. 3b). Urine production, measured via catheter, reached 1500 cm^3^/24 h (Fig. 3a). The outcome was good, with no noticeable complaints. Percutaneous drainage was removed 2 weeks after surgery after minimum production and the DJ stent was removed 1 month after surgery.

## 3 Discussion

Renal trauma often occurs in young males, with an incidence rate of 4.9 per 100,000 individuals. The leading cause of such trauma is blunt injury resulting from events like traffic accidents, collisions, falls, or assaults, which can lead to damage to the kidney or its hilum. A sudden deceleration force can cause an avulsion of the vascular structures in the hilum or the ureteropelvic junction (UPJ) [13]. Our case respectively discussed a young adult female involved in a motorcycle accident and presented with symptoms of grade IV renal trauma. It was managed with a DJ stent and percutaneous drainage.

Serial imaging for renal trauma often reveals fluid collections, which can manifest as hematomas, urinomas, or abscesses. Radiographic characteristics aid in distinguishing urinomas from hematomas. Urinomas typically exhibit a density range of 0 to 20 Hounsfield units (HU), whereas hematoma density is almost always >30 [14]. Urinomas demonstrate contrast pooling and enhancement during delayed phase imaging (5 to 20 min after intravenous contrast administration). On the other hand, abscesses display rim enhancement and high attenuation fluid (HU > 20) on contrast-enhanced images [15]. If perinephric fluid collections persist despite interventions such as ureteral stenting or percutaneous nephrostomy drainage, a percutaneous drain can facilitate healing and prevent or treat abscesses. In our specific case, the CT scan findings revealed the presence of hematomas.

Our study focused on young adults who had sustained untreated high-grade renal trauma and the presence of urinoma as a complication. Conservative or non-operative management is currently the standard of care for hemodynamically stable patients with AAST grade I to III renal injuries, irrespective of the causative mechanism [16]. Bluntly injured kidneys exhibit favorable healing outcomes when treated conservatively despite cases involving urinary extravasation and nonviable tissue. Approximately only 2 % of cases require exploration through surgical intervention. Non-operative management can even be effective for high-grade injuries in certain instances. A separate study revealed that patients experiencing life-threatening bleeding from the kidney may still necessitate exploration and subsequent nephrectomy/renorrhaphy. At the same time, hemodynamically stable individuals can often be treated successfully without surgery [17].

This case report shows the management of complications that arise (urinoma) in failed conservative therapy of high-grade renal trauma. Management of the complications that arise is done by installing a stent and percutaneous drainage to prevent urine leakage and remove the source of infection from the urinoma in the flank. Urology procedures such as ureteric stenting allow urine to be more easily drained from the kidney into the bladder by inserting a hollow tube into the kidney, ureter, and bladder. In a range of clinical situations, it is frequently used to relieve renal blockage and provide upper urinary tract drainage. A drainage catheter is typically inserted into the urinoma as first-line treatment, along with empirical antibiotics for relevant clinical signs and symptoms. A percutaneous nephrostomy tube, frequently combined with a ureteral stent to aid in healing, may be implanted to enable urinoma drainage in the event that the catheter is unable to empty the bladder sufficiently [18].

## 4 Conclusion

If conservative trauma fails and there are complications in cases of high-grade renal trauma, namely urinoma, urinary diversion needs to be performed, DJ stent insertion, and percutaneous drainage.

## Additional information

All information is private for this paper.

## Ethical approval

Ethical approval for this study was provided by Health Research Ethics Committee of Dr. Soetomo General-Academic Hospital, Surabaya.

## Funding

The author(s) received no financial support for the research.

## Author contribution

APA: Conceptualization, Methodology, Data Curation, Investigation, Writing-Original draft preparation.

AGPP: Conceptualization, Data Curation, Writing-Original draft preparation.

YAA: Data Curation, Writing original draft-Reviewing, and Editing.

KMS: Data Curation, Writing original draft-Reviewing, and Editing.

SW: Writing original draft, Reviewing, Supervision, Validation.

## Guarantor

Soetojo Wirjopranoto.

## Conflict of interest statement

The authors declare no conflict of interest.

## Data Availability

No data was used for the research described in the article.
